# Direct separation of minor actinides from high level liquid waste by Me_**2**_**-**CA-BTP/SiO_2_-P adsorbent

**DOI:** 10.1038/s41598-017-14758-2

**Published:** 2017-10-31

**Authors:** Shun Yan Ning, Xin Peng Wang, Qing Zou, Wei Qun Shi, Fang Dong Tang, Lin Feng He, Yue Zhou Wei

**Affiliations:** 1 School of Resources, Environment and Materials, Guangxi University, Nanning, 530004 China; 2 State Key Laboratory of Processing for Non-ferrous Metal and Featured Materials, Guangxi University, Nanning, 530004 China; 3 School of Nuclear Science and Engineering, Shanghai Jiao Tong University, Shanghai, 200240 China; 4 Shanghai Institute of Measurement and Testing Technology, Shanghai, 201203 China

## Abstract

Directly separating minor actinides (MA: Am, Cm, etc.) from high level liquid waste (HLLW) containing lanthanides and other fission products is of great significance for the whole nuclear fuel cycle, especially in the aspects of reducing long-term radioactivity and simplifying the post-processing separation process. Herein, a novel silica-based adsorbent Me_2_-CA-BTP/SiO_2_-P was prepared by impregnating Me_2_-CA-BTP (2,6-bis(5,6,7,8-tetrahydro-5,8,9,9-tetramethyl-5,8-methano-1,2,4-benzotriazin-3-yl)pyridine) into porous silica/polymer support particles (SiO_2_-P) under reduced pressure. It was found Me_2_-CA-BTP/SiO_2_-P exhibited good adsorption selectivity towards ^241^Am(III) over ^152^Eu(III) in a wide nitric acid range, acceptable adsorption kinetic, adequate stability against γ irradiation in 1 and 3 M HNO_3_ solutions, and successfully separated ^241^Am(III) from simulated 3 M HNO_3_ HLLW. In sum, considering the good overall performance of Me_2_-CA-BTP/SiO_2_-P adsorbent, it has great application potential for directly separating MA from HLLW, and is expected to establish an advanced simplified MA separation process, which is very meaningful for the development of nuclear energy.

## Introduction

As the development of nuclear energy, spent nuclear fuel in storage has amounted to around 266 000 t of heavy metal (HM) and is accumulating at a rate of around 7000 t HM/year, which has long-term potential radioactivity threats to the environment and must be managed safely and efficiently. Although the industrialized PUREX (Plutonium Uranium Recovery by EXtraction) process based on TBP can recover 99.5% of uranium and plutonium from spent nuclear fuel, the resulting high-level radioactive liquid waste (HLLW) containing most of the fission products (FP) and minor actinides (MA: Am, Cm, etc.) reserves most of the radioactivity, especially the α-emitters MA, which are the main contributors of radiotoxicity after three centuries storage^1^. Partitioning MA and the other long-lived FP from HLLW and then transmuting them into short-lived or stabile nuclides, which is the so-called Partition and Transmutation strategy (P&T), can reduce the time isolated from the environment from over 20,000 years to 300–500 years^2–5^. However, as the content of lanthanides (Ln), which accounts for about 1/3 of the FP, is much higher than that of MA in order of magnitude, and some Ln have large neutron absorption cross sections and are neutron poisons, Ln should be separated from MA before the transmutation step^5,6^. Considering MA and Ln dominantly exist as trivalent cations in solution with comparable radii and coordination numbers, mutual separation of MA(III) and Ln(III) is very difficult, which requires high selectivity for the separation materials^7–11^. Moreover, as the acid in HLLW produced by PUREX process was 2–5 M HNO_3_ and HLLW is highly radioactive with α, β, γ radioactivity, it further requires that the materials used for MA separation shall  have good acid and irradiation resistance stability.

**Figure 1:**
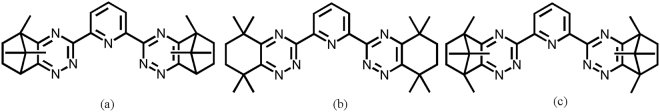
Chemical structures of (**a**) CA-BTP, (**b**) CyMe_4_-BTP and (**c**) Me_2_-CA-BTP.

Processes developed over the past decades are mainly based on the co-extraction of MA(III) and Ln(III) from HLLW followed by the An(III)/Ln(III) group separation^5,12,13^. Firstly, MA and Ln are co-separated from the other FP from high acid (mol/L) HLLW, e.g. the TRPO process using 30% (v/v) mixture of trialkyl phosphine oxides (TRPO) in kerosene^14^, the TRUEX process using [CMPO] = 0.2 M + [TBP] = 1.4 M in n-dodecane^12,15^, the DIAMEX process using DMDBTDMA or DMDOHEMA in HTP^1,5^ and the DIDPA process using 0.5 M DIDPA-0.1 M TBP in n-dodecane^16^. The oxygen donor ligands, e.g. CMPO, DMDOHEMA, TRPO and DIDPA mentioned above can separate MA(III) and Ln(III) together from fission products in HLLW, but exhibit poor adsorption selectivity between MA(III) and Ln(III). Secondly mutual group separation of MA and Ln are needed, making the integral HLLW separation process very complex. According to the hard and soft acid-base theory, MA(III) and Ln(III) both belong to hard acid, but MA(III) is a little softer than Ln(III) and has better coordination complexation with ligands containing soft coordination atoms N or S^17^. Using ligands containing N or S is expected to achieve the mutual group separation of MA(III) and Ln(III), e.g. BTP (BTBP) and CYANEX 301 used in SANEX^1,18^, CYANEX 301 processes^19^ respectively. Among the various N or S donor ligands, 2,6-bis(5,6-dialkyl-1,2,4-triazin-3-yl)pyridines (BTPs) and 6,6’-bis-(5,6-dialkyl-1,2,4-triazin-3-yl)-2,2’-bipyridines (BTBPs) both exhibit high selectivity for MA(III) over Ln(III) in a wide range of nitric acid solutions (HNO_3_: 0.1–4 M, M = mol/L) with the separation factor *SF*_Am/Eu_ around 100 and are considered to be a significant breakthrough in MA separation as before that separation MA could be realized only in low acid (pH range)^20–26^. Furthermore, BTPs/BTBPs contain only C, H, N elements and fulfill the CHON principle and thus are completely combustible to gaseous products after use, reducing the second radioactive waste. However, the presently available BTPs used in liquid-liquid extraction suffer from one or more drawbacks, e.g. poor solubility property in traditional solvents (e.g. *i-*Pr-BTP, CyMe_4_-BTBP)^1,26^, slow stability against acid and irradiation (e.g. n-alkylated BTPs and BTBPs)^26–28^, low loading capacity (e.g. CA-BTP)^28^, and poor back extraction performance (e.g. CyMe_4_-BTP)^26^, slow kinetic^26^ etc. To improve it, We synthesized a new kind of BTP, Me_2_-CA-BTP (2,6-bis(5,6,7,8-tetrahydro-5,8,9,9-tetramethyl-5,8-methano-1,2,4-benzotriazin-3-yl) pyridine) based on the structure of both CyMe_4_-BTP and CA-BTP avoiding -CH_2_- group on the α position of the triazine rings which is the weak spot of the ligand under degradation to make it more stable with their chemical structures shown in Fig. 1 ^26,29,30^.

In a word, more challenging processes for directly separating MA from HLLW are eagerly needed to be developed. Separating MA from HLLW by one step process has been proposed, such as 0.2 M CMPO + 1.4 M TBP in *n*-dodecane used in SETFICS process^5^, 0.015 M CyMe_4_-BTBP + TODGA or DMDOHEMA in TPH/Octanol used in the 1-cycle SANEX process^5,13^, TODGA + water-soluble SO_3_-Ph-BTBP in TALSPEAK process^31^. As the processes mentioned above involved more than one kind of extractant, it is inconvenient for the organic phase regeneration. Wei. etc. proposed an compact and effective process based on extraction chromatographic technology named MAREC process (Minor actinide extraction by chromatographic process) as shown in Fig. 1S ^32^ whose purpose is to directly separate MA(III) from PUREX raffinate HLLW containing fission products Ln and other FPs through single column packed with high-efficiency microporous adsorbent, e.g., BTPs/SiO_2_-P. If success, it will significantly simplify the MA separation process and is of great significance for the whole nuclear fuel cycle. Efforts to realize the single-column MAREC process from simulated HLLW have been made by using isoHex-BTP/SiO_2_-P by co-authors^33,34^, but isoHex-BTP/SiO_2_-P turned out to be easy to lose the adsorption ability when it accepted γ irradiation in 3 M HNO_3_ solution in our previous research^35^, so more advanced materials are needed to be developed. Furthermore, MAREC process uses almost no organic solvent, avoiding the third phase formation and large amount of secondary organic waste accumulation.

In this work, Me_2_-CA-BTP/SiO_2_-P adsorbent was prepared by impregnating 5 g Me_2_-CA-BTP dissolved in CH_2_Cl_2_ into 10 g stable microporous silica/polymer composite support (SiO_2_-P) particles under reduced pressure. The adsorbent properties of selectivity, kinetics, γ-irradiation stability was studied by batch adsorption experiments. Finally, the feasibility of using Me_2_-CA-BTP/SiO_2_-P adsorbent to directly separate MA(III) from 3 M HNO_3_ simulated HLLW through single-column MAREC process was evaluated.

## Results

### Adsorption selectivity

**Figure 2:**
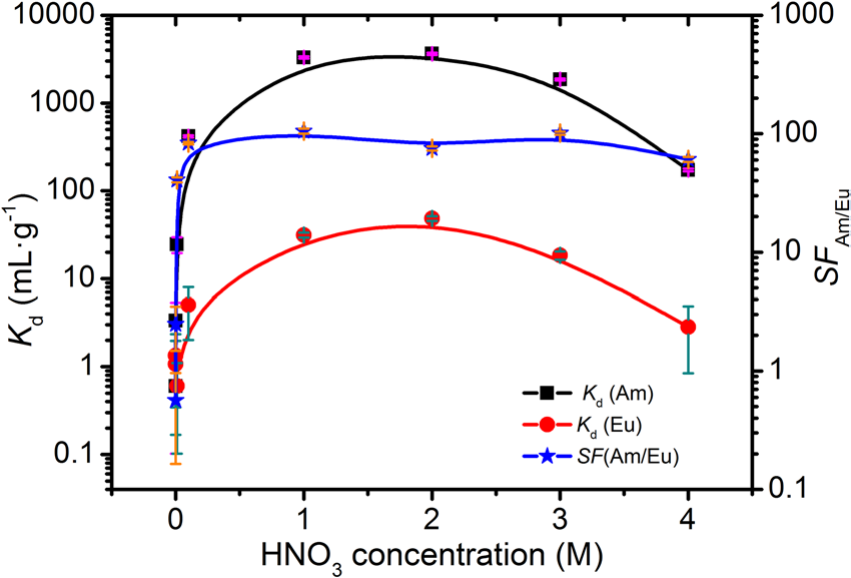
Effects of HNO_3_ concentration on Me_2_-CA-BTP/SiO_2_-P adsorption towards ^241^Am(III) and ^152^Eu(III) (phase ratio: 0.1 g/5 mL, ^241^Am(III): 1000 Bq·mL^−1^ (3.27*10^−8^ mol/L), ^152^Eu(III): 1000 Bq·mL^−1^ (1.02*10^−9^ mol/L), Temp.: 25 °C, contact time: 24 h, shaking speed: 300 rpm).

To study adsorption selectivity, the effects of initial HNO3 concentration (pH = 4–4 M) on Me_2_-CA-BTP/SiO_2_-P adsorption towards ^241^Am(III) and ^152^Eu(III) were evaluated. Figure 2 illustrates the distribution coefficients (*K*_d_) of ^241^Am(III) and ^152^Eu(III) and the separation factors of ^241^Am(III) over ^152^Eu(III) (*SF*_Am/Eu_) changing as a function of HNO_3_ concentration. The distribution factors *K*_d_ of ^241^Am(III) and ^152^Eu(III) increased as the HNO_3_ concentration increased until up to 2 M HNO_3_ solution which are explained by the adsorption of BTP towards ^241^Am(III) needing the participation of NO_3_^−^^28^. Then with further increase of the acid, the distribution factors *K*_d_ of ^241^Am(III) and ^152^Eu(III) decreased. The decreasing distribution factors are explained by metal ions (^241^Am(III) and ^152^Eu(III)) and nitric acid competing adsorption for BTP. The whole adsorption mechanism may be as shown in Equation (1) and (2) similar to CA-BTP^28^. Three NO_3_^−^ complexes are incorporated into the coordination sphere so as to maintain neutral due to metal iron’s trivalent oxidation state

1 $${{\rm{M}}}^{3+}+{{\rm{3NO}}}_{{\rm{3}}}^{-}+3{{\rm{Me}}}_{{\rm{2}}}{-{\rm{CA}}-{\rm{BTP}}/{\rm{SiO}}}_{{\rm{2}}}-{\rm{P}}\to {\rm{M}}{({{\rm{Me}}}_{{\rm{2}}}{-\mathrm{CA}-\mathrm{BTP}/\mathrm{SiO}}_{{\rm{2}}}-{\rm{P}})}_{3}{{(\mathrm{NO}}_{3})}_{3}$$.

2 $${{\rm{H}}}^{+}+{{\rm{NO}}}_{{\rm{3}}}^{-}+{{\rm{Me}}}_{{\rm{2}}}{-{\rm{CA}}-{\rm{BTP}}/{\rm{SiO}}}_{{\rm{2}}}-{\rm{P}}\to {{\rm{Me}}}_{{\rm{2}}}{-{\rm{CA}}-{\rm{BTP}}/{\rm{SiO}}}_{{\rm{2}}}-{\rm{P}}\cdot {{\rm{HNO}}}_{3}$$

Overall, Me_2_-CA-BTP/SiO_2_-P exhibited good adsorption selectivity towards ^241^Am(III) with the uptake rate of ^241^Am(III) over 96% and *SF*_Am/Eu_ over 75 in a wide nitric acid range of 1–3 M, which was high enough for effective separation ^241^Am(III) from ^152^Eu(III). Furthermore, according to previous work^29,36^, Me_2_-CA-BTP/SiO_2_-P showed almost no adsorption ability towards most typical FP except Pd(II) in 3 M HNO_3_. The results above indicates that Me_2_-CA-BTP/SiO_2_-P has the potential to directly separate MA from high nitric acid (3 M) HLLW. The adsorption ability and the applicable acidity range of Me_2_-CA-BTP/SiO_2_-P towards Am(III) has been improved and enlarged compared with CA-BTP which only exhibited good adsorption ability towards Am(III) in 0.5–1 M HNO_3_ and CyMe_4_-BTBP whose adsorption ability towards Am(III) increased as HNO_3_ concentration increased in 0.1–2 M HNO_3_ and isoHex-BTP which only exhibited good adsorption towards ^241^Am(III) in ≥ 2 M HNO_3_ solution^28,37,38^.

### Adsorption kinetics

**Figure 3:**
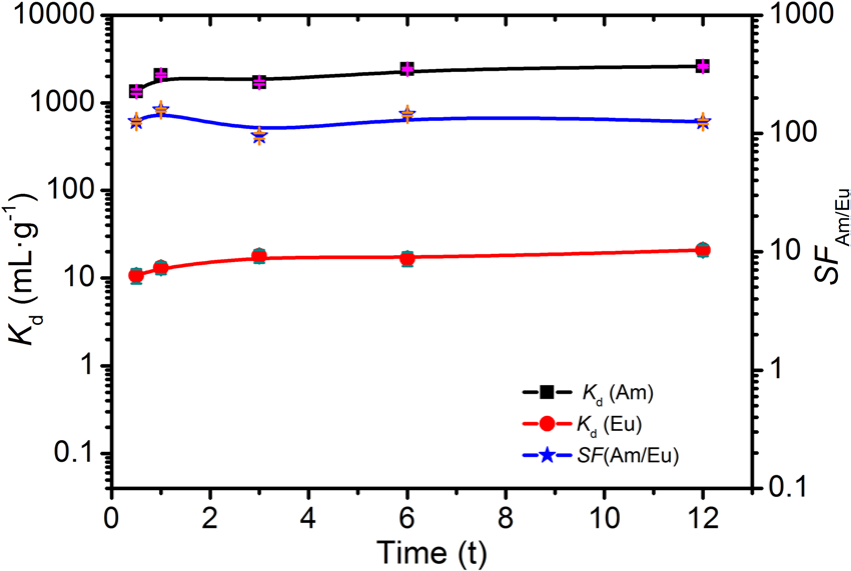
Adsorption kinetics of ^241^Am(III) and ^152^Eu(III) on Me_2_-CA-BTP/SiO_2_-P in 1.0 M HNO_3_ solution (phase ratio: 0.1 g/5 mL, ^241^Am(III): 1000 Bq·mL^−1^ (3.27*10^−8^ mol/L), ^152^Eu(III): 1000 Bq·mL^−1^ (1.02*10^−9^ mol/L), Temp.: 25 °C, shaking speed: 120 rpm).

The effect of contact time on Me_2_-CA-BTP/SiO_2_-P adsorption towards ^241^Am(III) and ^152^Eu(III) was studied in 1.0 M HNO_3_ solution at 25 °C with the results shown in Fig. 3. Evidently, Me_2_-CA-BTP/SiO_2_-P adsorbed ^241^Am(III) and ^152^Eu(III) quickly reaching almost equilibrium within 0.5 h and exhibited much higher adsorption affinity towards ^241^Am(III) than ^152^Eu(III) with *SF*_Am/Eu_ around 100. Considering the equilibrium time for CA-BTP and CyMe_4_-BTBP adsorption towards Am(III) was about 10 min and 30 min in solvent extraction^27,28^, the three systems mentioned above have comparable adsorption kinetic.

### Stability evaluation

**Figure 4:**
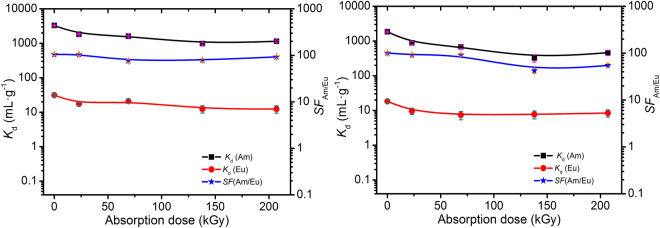
Effect of γ irradiation absorbed dose in (a) 1 M HNO_3_ (b) 3 M HNO_3_ on Me_2_-CA-BTP/SiO_2_-P adsorption towards ^241^Am(III) and ^152^Eu(III) in (a) 1.0 M (b) 3 M HNO_3_ solution (irradiation conditions: ^60^Co-γ source, 1 kGy/h, phase ratio: 0.8 g/40 mL; adsorption conditions: phase ratio: 0.1 g/5 mL, Temp.: 25 °C, contact time: 24 h, ^241^Am(III): 1000 Bq·mL^−1^ (3.27*10^−8^ mol/L), ^152^Eu(III): 1000 Bq·mL^−1^ (1.02*10^−9^ mol/L), shaking speed: 120 rpm).

Considering the radioactive nuclides in HLLW emit vast amounts of radiation, e.g., α, β, γ, the adsorbent irradiation stability is a serious concern in practical applications for HLLW reprocessing. Herein, the stability of Me_2_-CA-BTP/SiO_2_-P adsorbent against γ-irradiation in 1 and 3 M HNO_3_ solution were evaluated. 0.8 g adsorbent was combined with 40 mL 1 or 3 M HNO_3_ solution in 50 mL glass vial and irradiated by ^60^Co-γ source with the absorbed radiation dose rate of 1 kGy·h^−1^. After being irradiated to the predetermined doses, vacuum filter was used for solid-liquid separation. After drying the Me_2_-CA-BTP/SiO_2_-P adsorbent in room temperature, the Me_2_-CA-BTP/SiO_2_-P adsorbent was used to adsorbe ^241^Am(III) and ^152^Eu(III) in 1 and 3 M HNO_3_ solution respectively with the results shown in Fig. 4. The *K*_d_ of ^241^Am(III) decreased as the absorbed dose increased in both 1 M and 3 M HNO_3_ solution but was still kept in a high level over 983 and 325 mL/g respectively when the absorbed dose was up to 207 kGy. Meanwhile Me_2_-CA-BTP/SiO_2_-P adsorbent maintained good adsorption selectivity towards ^241^Am(III) over ^152^Eu(III) with *SF*_Am/Eu_ over 76 and 42 in 1 M and 3 M HNO_3_ solution respectively in the whole irradiation experiments. Furthermore, considering when isoHex-BTP was irradiated in 3 M HNO_3_ under the same irradiation conditions, it quickly lost the adsorption ability^35^, and CyMe_4_-BTBP distribution factors of ^241^Am(III) decreased from about 22 to about 12 in 1 M HNO_3_ when the absorbed γ dose reached 200 kGy with the absorption dose rate of 0.22 kGy/h^22^, Me_2_-CA-BTP/SiO_2_-P adsorbent exihibted very good γ irradiation stability in 1 M and 3 M HNO_3_ solution and are expected to directly separate MA from high nitric acid HLLW.

### Directly separate MA(III) from HLLW by single-column MAREC process

**Figure 5:**
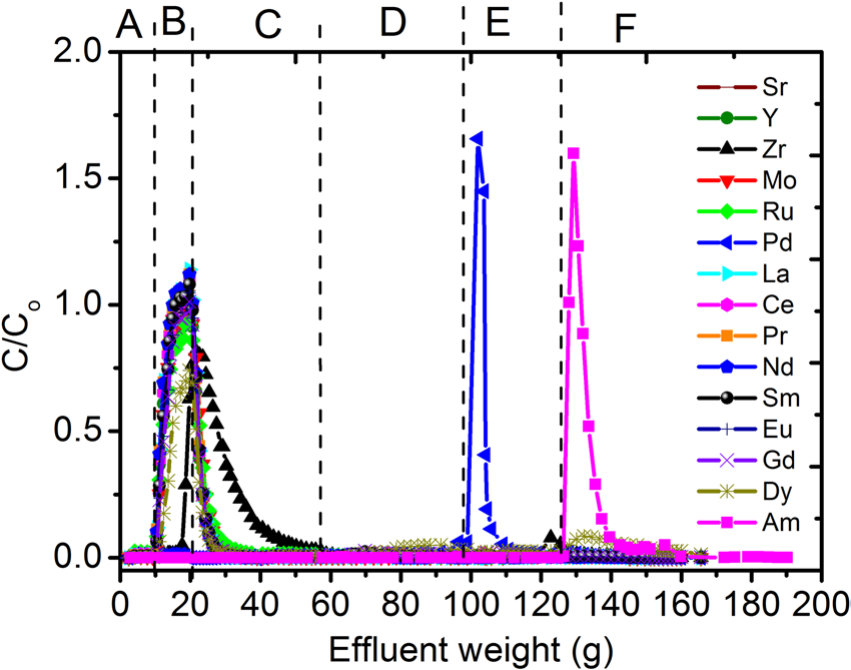
Directly separate MA(III) from simulated 3 M HNO_3_ HLLW through single-column MAREC process (Temp.: 35 °C, liquid flow rate: about 0.1 mL/min, A: 3 M HNO_3_, B: 3 M HNO_3_, 5 mM [M], 500 Bq/mL ^241^Am(III), C: 3 M HNO_3_, D: 0.1 M HNO_3_, E: 0.01 M HNO_3_ -0.1 M thiourea, F: 0.001 M HNO_3_-0.01 M DTPA).

To examine the separation of MA(III) from fission products, a hot test using simulated 3 M HNO_3_ HLLW containing typical FP (Sr, Y, Zr, Mo, Ru, Pd, La, Ce, Nd, Sm, Eu, Gd, Dy, 5 mM respectively) and ^241^Am(III) (500 Bq/mL) was carried out at 35 °C using a glass column (5 mm in inner diameter and 500 mm in length) packed with 5 g Me_2_-CA-BTP/SiO_2_-P with the results shown in Fig. 5 and Table 1. As can be seen, Me_2_-CA-BTP/SiO_2_-P showed very poor or almost no adsorption towards lanthanides and most other typical fission products, such as Sr, Y, Zr, Mo, Ru. These elements flowed out with the feed solution in step B and the following 3 M and 0.1 M HNO_3_ solution in step C and D. On the other hand, Pd, well known as a “soft” metal ion which has strong electron acceptance ability, was adsorbed onto Me_2_-CA-BTP/SiO_2_-P and could not be effectively eluted from the adsorbent by reducing the HNO_3_ concentration. A complexing agent, thiourea, which has strong complexation affinity with Pd, was attempted to desorb the adsorbed Pd. As shown in Fig. 5, Pd can be effectively eluted off by 0.01 M HNO_3–0.1_ M thiourea in step E. Meanwhile, ^241^Am was strongly adsorbed by Me_2_-CA-BTP/SiO_2_-P adsorbent, then it was eluted off using 0.001 M HNO_3_-0.01 M DTPA as an eluent with ^241^Am recovery yield of 95.87% and the other typical fission products less than 3% except Dy. But considering there is almost no Dy or other Ln heavier than Dy in HLLW, the effects caused by Dy are almost negligible. In a word, a successful separation between Am and the various typical fission products including lanthanides has been achieved, which is an important step toward a simplified direct process for separation MA from HLLW.

**Table 1:** Recovery yields of tested elements with Me_2_-CA-BTP/SiO_2_-P adsorbent.

Recovery yield (%)	B (%)	C (%)	D (%)	E (%)	F (%)	Total recovery yield (%)
Sr	87.2	9.3	1.8	0.9	0.8	99.9
Y	82.5	10.1	3.2	1.8	2	99.8
Zr	23.5	72.5	0.9	0.9	6.7	104.5
Mo	82.2	15	1.1	1.1	1.7	101.1
Ru	77.5	18.9	2.4	1.1	1.7	101.5
Pd	1.6	1.5	7.3	78.6	2.6	91.6
La	93.2	9.1	0.6	0.2	0.4	103.4
Ce	89.6	9.7	2.3	1.3	0.9	103.8
Pr	92.2	9.8	1.6	0.8	0.7	105.2
Nd	92.4	9.6	0.7	0.1	0.3	103
Sm	86.2	10.5	3.1	1.2	1.6	102.6
Eu	80.6	11.2	4.5	1.8	1.5	99.8
Gd	77.9	11.1	5.5	2.6	2.8	99.9
Dy	50.3	8.5	11	4.5	19.4	93.7
Am	0	0.03	0.13	0.01	95.87	96.04

## Discussion

To directly separate long-lived MA from HLLW, a single-column MAREC process was proposed and a corresponding novel microporous silica-based Me_2_-CA-BTP/SiO_2_-P adsorbent was prepared with the content of Me_2_-CA-BTP as high as 32 wt%. The research found Me_2_-CA-BTP/SiO_2_-P exhibited good adsorption selectivity towards ^241^Am(III) with the uptake rate of ^241^Am(III) over 96% and *SF*_Am/Eu_ over 75 in a wide nitric acid range of 1–3 M. In 1 M HNO_3_ solution, the adsorption of Me_2_-CA-BTP/SiO_2_-P towards ^241^Am(III) almost reached equilibrium within an hour with the uptake rate of 96% showing rapid adsorption kinetic. With the increase of γ irradiation absorbed dose in 1 M HNO_3_ and 3 M HNO_3_ solution, the adsorption performance of Me_2_-CA-BTP/SiO_2_-P towards ^241^Am(III) did not change significantly. When γ irradiation absorbed dose reached to 207 kGy, Me_2_-CA-BTP/SiO_2_-P maintained good adsorption selectivity towards ^241^Am(III), indicating Me_2_-CA-BTP/SiO_2_-P has good resistance to γ irradiation stability. Furthermore, the continuous column separation experimental results showed that Me_2_-CA-BTP/SiO_2_-P can effectively and selectively separate ^241^Am(III) from the other fission products from simulated 3 M HNO_3_ HLLW with ^241^Am(III) recovery rate of 95.5% in ^241^Am(III) elution step.

In a word, Me_2_-CA-BTP/SiO_2_-P has great application potential in the single-column MAREC process, and is expected to establish an advanced simplified MA(III) separation process.

## Methods

### Reagents

FP element nitrates (FP: Sr(II), Zr(IV) and trivalent rare earths) and (NH_4_)_6_Mo_7_O_24_•4H_2_O were commercial reagents of analytical grade. Pd(NO_3_)_2_•2H_2_O was chemical pure with Pd(II) ≥ 39.5 wt% and chloridate ≤ 0.04 wt%. Ru(III) nitrosyl nitrate solution was in diluted nitric acid containing 1.5 wt% of Ru(III) with a density of 1.07 g·mL^−1^. ^241^Am(III) and ^152^Eu(III) were from laboratory stock solution. Me_2_-CA-BTP was synthesized at laboratory with the purity of 97% according to the HPLC-MS test. All solutions were prepared with deionized water at 18 MΩ·cm resistance (DI water). Other agents such as nitric acid, dichloromethane, etc. were of analytical grade and used without further treatment.

### Preparation of Me_2_-CA-BTP/SiO_2_-P adsorbent

The support SiO_2_-P with pore size of 0.6 μm, pore fraction of 0.69 and mean diameter of 50 μm was developed in the previous work^39^. P refers to macroreticular styrene-divinylbenzene copolymer (SDB) and is immobilized in porous silica (SiO_2_) particles with the content of 17–18 wt% in SiO_2_-P. The synthesis procedure of Me_2_-CA-BTP/SiO_2_-P adsorbent was the same as ref.^40^. The synthesized Me_2_-CA-BTP/SiO_2_-P adsorbent overcame the limitation of low solubility of BTP in traditional diluents as the content of Me_2_-CA-BTP was as high as 32.0 wt% in the adsorbent^29^. The synthetic Me_2_-CA-BTP/SiO_2_-P adsorbent was characterized by high resolution field emission scanning electron microscope (SEM, Sirion 200, FEI COMPANY) and the SEM image is shown in Fig. 2S.

### Batch adsorption experiments

For batch adsorption experiments towards ^241^Am(III) and ^152^Eu(III), 0.1 g adsorbent was combined with 5 mL aqueous solution in a 12 mL glass vial with screw teflon cap. The mixture in the vial was shaken mechanically at 300 rpm at 25 °C for a certain time and the solid-liquid separation was realized by centrifugation. The radioactivity of ^241^Am(III) and ^152^Eu(III) in solution before and after adsorption were determined by high-purity germanium multichannel gamma spectrometer (CANBERRA) at 59.5 and 121.78 keV respectively, while the concentrations of non-radioactive FP were determined by inductively coupled plasma-optical emission spectrometer atomic emission spectroscopy (ICP-AES: Shimadzu ICPS-7510).

The distribution coefficient *K*_d_ (mL·g^−1^), separation factor *SF*_A/B_ and uptake rate *E* (%) which are key factors in solid-liquid adsorption are calculated by Equation (3), (4) and (5), respectively.

3 $${K}_{{\rm{d}}}=\frac{{A}_{{\rm{o}}}\,-\,{A}_{{\rm{e}}}}{{A}_{{\rm{e}}}}\times \frac{V}{W}$$

4 $$S{F}_{{\rm{A}}/{\rm{B}}}={K}_{{\rm{d}}{\rm{A}}}/{K}_{{\rm{d}}{\rm{B}}}$$

5 $$E\,=\,\frac{({A}_{{\rm{o}}}\,-\,{A}_{{\rm{e}}})}{{A}_{{\rm{o}}}}\times 100 \% $$

where *A*_o_, *A*_e_ denote the radioactivity of metal ions in the aqueous phase before and after adsorption, respectively, Bq·mL^−1^. *V* (mL) indicates the volume of aqueous phase and *W* (g) is the mass of dry Me_2_-CA-BTP/SiO_2_-P.

### Column separation experiments

Column separation experiments for simulated HLLW solution were carried out using a glass column with 5 mm in diameter and 500 mm in length. 5 g Me_2_-CA-BTP/SiO_2_-P adsorbent was transferred to the column in the slurry state under atmosphere. The column volume (CV) of the adsorbent bed was 9.8 cm^3^. The column was kept at a constant temperature (35 °C) with water jacket. Prior to the separation experiment, the adsorbent in column was pre-equilibrium by passing 50 mL of 3 M HNO_3_. The simulated HLLW contained 5 m mol/L for each FP (Sr, Y, Zr, Mo, Ru, Pd, La, Ce, Pr, Nd, Sm, Eu, Gd, Dy) and 500 Bq/mL ^241^Am(III) in 3 M HNO_3_ solution. All the mobile phase was pumped at 0.1 mL/min and the effluents were collected by a fractional collector. The metal concentrations in the effluents without radioactivity were determined by ICP-AES while ^241^Am(III) was determined by high-purity germanium multichannel gamma spectrometer.

### Statistical analysis

Each batch adsorption experiment was conducted in double parallel, and batch adsorption values used are means ± SD.

### Data availability

The authors declare that all data supporting the findings of this study are available within the article and its Supplementary Information files.

## Electronic supplementary material

Supplementary information
