# Supplementary material for: Direct separation of minor actinides from high level liquid waste by Me2-CA-BTP/SiO2-P adsorbent
Source: Sci Rep. 2017 Oct 31;7:14679. doi: 10.1038/s41598-017-14758-2 (PMC5665906; doi:10.1038/s41598-017-14758-2)
Supplement: Supplementary file 1 — Supplementary information [file 41598_2017_14758_MOESM1_ESM.pdf]

Direct separation of minor actinides from high level liquid waste by Me_2-CA-BTP/SiO_2-P adsorbent

Shun Yan Ning[1,2], Xin Peng Wang[1,2], Qing Zou[3], Wei Qun Shi[1], Fang Dong Tang[4], Lin Feng He[4], Yue Zhou Wei*[1,2,3]

1. School of Resources, Environment and Materials, Guangxi University, Nanning, 530004, China;

2. State Key Laboratory of Processing for Non-ferrous Metal and Featured Materials, Guangxi University, Nanning, 530004, China; 3. School of Nuclear Science and Engineering, Shanghai Jiao Tong University, Shanghai, 200240, China; 4. Shanghai Institute of Measurement and Testing Technology, Shanghai, 201203, China

*Corresponding author: YueZhou Wei

Telephone: +86-0771-3224990

Fax: +86-0771-3224990

E-mail: yzwei@gxu.edu.cn

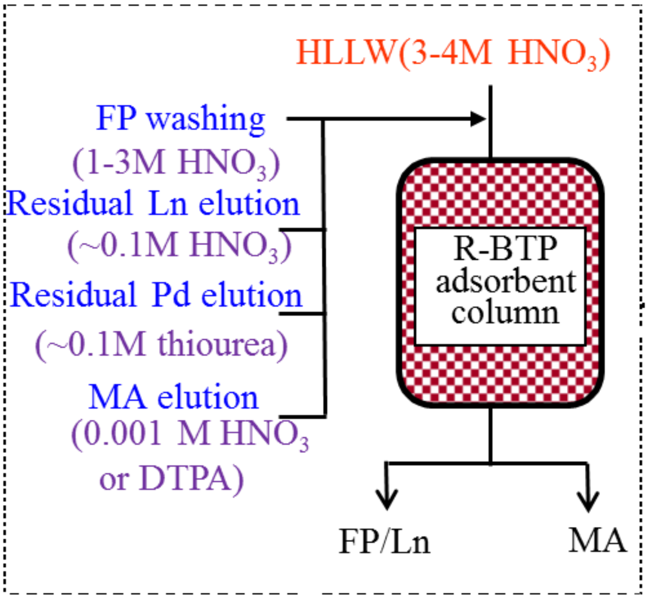

Fig. S1 Flowsheet of single-column MAREC process

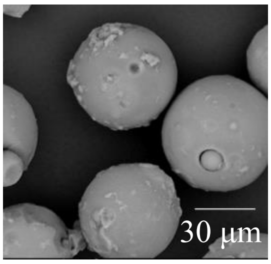

Fig. S2 SEM image of Me_2-CA-BTP/SiO_2-P
